# Investigation of Phenolic Composition and Antioxidant Capacities in Selected Turkish Indigenous Wheat Varieties

**DOI:** 10.1002/fsn3.4614

**Published:** 2025-01-31

**Authors:** Muhammad Usman Akram, Ayşegül Bilge Uğuz, Umran Uygun, Ayten Salantur, Remziye Yilmaz

**Affiliations:** ^1^ Faculty of Engineering, Department of Food Engineering Hacettepe University Ankara Turkey; ^2^ Ministry of Agriculture and Forestry Field Crops Central Research Institute Ankara Turkey

**Keywords:** antioxidant capacity, flavonoids, indigenous wheat, phenolic acids

## Abstract

Indigenous wheat varieties continue to be cultivated and preserved due to their climatic adaptability and distinctive flavor contributions to cultural cuisines. This study covers the research gap by investigating the natural bioactive and antioxidant compounds present in Turkish indigenous wheat varieties. To this aim, the phenolic composition, ash contents, and antioxidant capacity were investigated in eighteen different indigenous Turkish wheat varieties (4 monococcum, 3 dicoccum, 4 durum, and 7 aestivum genotypes). An overall comprehensive analysis was carried out using statistical tools such as Heatmap and PCA (Principal component analysis). The results indicated that dicoccum genotype exhibited highest soluble free (49.04 mg) and soluble conjugated phenolics (188.54 mg), whereas aestivum was found rich in insoluble bound (873.89 mg), total phenolic (1058.35 mg GAE/kg dm), and total flavonoid (380.43 mg CE/kg dm) contents. Moreover, monococcum, dicoccum, and durum genotypes exhibited non‐significantly higher total antioxidant capacities compared to the aestivum wheat genotype. In conclusion, indigenous wheat varieties, particularly from the dicoccum and aestivum genotypes, have the potential to be a significant source of phenolic and antioxidant compounds. These findings suggest promising prospects for the selection of indigenous wheat varieties under on‐farm conservation programs for future commercialization and breeding programs.

## Graphical abstract

The study investigated the phenolic composition, ash contents, and antioxidant capacities of 18 Turkish indigenous wheat varieties. Results showed that dicoccum genotypes have the highest soluble free and conjugated phenolics, while aestivum is rich in insoluble bound, total phenolic, and total flavonoid contents. Moreover, monococcum and dicoccum wheat genotypes have slightly higher total antioxidant capacities. These findings suggested potential for selection of indigenous wheat varieties under on‐farm conservation programs for future commercialization and breeding programs.

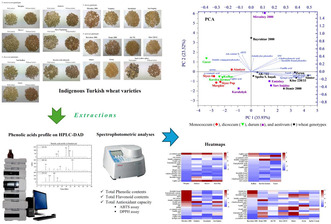

## 1 Introduction

Anatolian region of Turkey is home to more than 400 cultivated and around 23 wild wheat varieties (Turkyilmaz Unal 2020). The cultivation of wheat has undergone a process of selection, driven by both natural factors and the choices made by farmers. As a result, wheat varieties now exhibit a broader range of genetic variability, which makes them highly attractive for breeding programs (Kan et al. 2015). Wheat varieties are classified into two main genera (Triticum and Aegilops) and three ploidy levels, that are, diploid (2*n* = 14), tetraploid (2*n* = 28), and hexaploid (2*n* = 42), based on differences in chromosome number (Özberk et al. 2016). The implementation of modernization in agriculture through the Green Revolution together with the adoption of conventional breeding techniques aimed at achieving high yields has resulted in a significant reduction in genetic varieties (Sthapit et al. 2022). However, various evolutionary developments in winter wheat varieties have been observed with improvement of grain weight and yield‐related traits but with reduced plant heights (Wang et al. 2022). This also necessitates the development of the artificial guidelines through deep learning approaches, for the selection and classification of new wheat varieties (Laabassi et al. 2021).

There is growing interest in investigating indigenous wheat varieties because of their high tolerance toward biotic and abiotic stress conditions. These varieties also possess the potential to produce consistent or even higher crop yield compared to modern commercial types, especially in regions with limited resources and less favorable conditions. This attribute makes them highly valuable for the preservation of biodiversity and provides a practical alternative for addressing cultural markets (Preiti et al. 2022). Although indigenous wheat varieties such as einkorn ( *T. monococcum* ) and emmer ( *T. dicoccum* ) may have lower yields than modern varieties, their sustainability and higher nutritional profiles have garnered significant interest from both farmers and researchers. Additionally, the indigenous wheat varieties under on‐farm conservation are recognized for their bioactive compounds, which offer notable health benefits (Kan et al. 2015; Lo Bianco et al. 2017; Zhang et al. 2012). The bioactive compounds such as phenolic acids and flavonoids are synthesized through the phenylpropanoid and flavonoid pathways in whole wheat grain and typically present in various forms, including soluble free, soluble conjugated, and insoluble bound phenolic compounds (Feng et al. 2022). The conjugated and bound phenolic acids are primarily ester‐linked to sugar molecules and cell wall components, such as polysaccharides and lignin. Therefore, alkaline hydrolysis can prove to be an effective method for accurate quantification of phenolic compounds due to its ability to break the ester bonds (Hefni, Amann, and Witthöft 2019). These phenolic and flavonoid compounds are also considered as major antioxidants in wheat and have the ability to suppress lipid peroxidation by scavenging free radicals such as hydroxyl, peroxyl, and reactive oxygen species (Žilić et al. 2012).

Previous research works have proven that wheat varieties are believed to have significant sources of healthy phenolic compounds. However, there is a research gap in investigating the presence and quantification of phenolic acid composition and antioxidant contents in the indigenous Turkish wheat varieties. This aspect needs to be investigated further for the enhancement of the economic potential as well as for the assessment of their suitability in future breeding initiatives.

Therefore, the aim of this study is to investigate the presence of phenolic contents including soluble (free and conjugated), insoluble bound, total phenolics with their individual phenolic acid composition, total flavonoids, and ash contents in the indigenous wheat varieties. Additionally, the study also investigates the antioxidant capacity by quantifying the ability to neutralize free radicals using ABTS and DPPH assays. Heatmap and PCA biplot analyses were used to develop correlations between phenolic and antioxidant compounds of studied wheat varieties.

## 2 Materials and Methods

### 2.1 Research Materials

The research material consists of four *T. monococcum* , three *T. dicoccum* , four *T. durum* , and seven *T. aestivum* wheat genotypes, which were provided by the Field Crops Central Research Institute (TAGEM), Ankara, Turkey (Table 1). The images of Turkish indigenous wheat varieties (with and without hulled) are provided in Figure S1. These varieties have been selected due to their rich phenotypic characteristics (grain size, color, etc.) and their presence in on‐farm conservation program. All wheat genotypes were cultivated at Gölbaşı‐İkizce Research and Production Farm, Ankara (longitude 32.63 E and latitude 39.57 N) during the field studies carried out in 2019–2020, followed from their collection from the local farmers. In the 2019–2020 growth period; plantings were made in October 2019. The rainfall and temperature received in October, November, and December 2019 were at a level that would provide germination and emergence. In addition, good rainfall in April and May 2020 ensured that the yield was at the level of normal years. The standard agronomic climatic conditions for 2019–2020 growth period are provided in Table S1. The agriculture land of cultivation has an altitude of 1200 m above sea level, whereas the average annual precipitation and temperature in that territory remained at 377 mm and 11°C, respectively (NASA 2024). The soil has a slightly alkaline pH with a calcareous and clayey loamy texture, sufficient in potassium, but poor in organic matter, salt, phosphorus, iron, and zinc contents. Phosphorus fertilizer (diammonium phosphate) was given at planting period (6 kg P_2_O_5_/da), but nitrogenous fertilizer (ammonium nitrate) was applied both at planting (3 kg N/da) and tillering periods (3 kg N/da). All wheat seeds were planted under the same climatic conditions and the field using above‐mentioned standard agronomic practices.

**TABLE 1:** List of wheat genotypes selected from on‐farm conservation program.

Genotypes	Botanical name	Status	Origin
Mergüze	*Triticum monococcum* L. *ssp*. *monococcum*	Commercial^b^	Kastamonu
Atasiyez	*T. monococcum* L. *ssp*. *monococcum*	Commercial^b^	Kastamonu
Siyez‐4	*T. monococcum* L. *ssp*. *monococcum*	Pure line	Kastamonu
Siyez population	*T. monococcum* L. *ssp*. *monococcum*	Local	Kastamonu
Yeni Kafkas	*Triticum turgidum* L. *ssp. dicoccum*	Commercial^b^	Ardahan
Kavılca (red colored)	*T. turgidum* L. *ssp. dicoccum*	Pure line	Kars
Gacer	*T. turgidum* L. *ssp. dicoccum*	Local	Kayseri
Mirzabey 2000	*Triticum turgidum* L. *ssp. durum*	Commercial^a^	Central Anatolia
Eminbey	*T. turgidum* L. *ssp. durum*	Commercial^a^	Central Anatolia
Karakılçık	*T. turgidum* L. *ssp. durum*	Local	Hatay
Sarı bugday	*T. turgidum* L. *ssp. durum*	Local	Eskişehir
Bayraktar 2000	*Triticum aestivum* L. *ssp. aestivum*	Commercial^a^	Central Anatolia
Demir 2000	*T. aestivum* L. *ssp. aestivum*	Commercial^a^	Central Anatolia
AK‐702	*T. aestivum* L. *ssp. aestivum*	Local	Eskişehir
Köse 220/33	*Triticum aestivum* L. *ssp. vulgare*	Local	Sivas, Erzurum
Sünter	*T. aestivum* L. *ssp. vulgare*	Local	Eastern Anatolia
Zerun	*T. aestivum* L. *ssp. vulgare*	Local	Sivas, Erzurum
Spelt (yellow spike)	*Triticum aestivum* L. em Thell *spelta*	Pure line	Central Research Institute for Field Crops, Ankara

^a^ Initially chosen as commercial controls.

^b^ During research, some local wheat varieties were registered as commercial varieties.

Four commercial wheat varieties such as Mergüze, Yeni Kafkas, Eminbey, and Demir 2000 were used as controls to assess the quality parameters of indigenous varieties.

- Mergüze ( *Triticum monococcum* L.) is an einkorn wheat, with 2*n* = 14 chromosomes, a hulled wheat cultivar with light colored awn and spike, late, medium height, resistant to lodging.
- Yeni Kafkas ( *Triticum dicoccum* L.) is an emmer wheat, with 2*n* = 28 chromosomes, a hulled wheat cultivar with early, medium‐short, white awn and spike color.
- Eminbey ( *Triticum durum* ) is a durum wheat, with 2*n* = 28 chromosomes, white spikes, winter growth nature, and very good pasta quality.
- Demir 2000 ( *Triticum aestivum* ) is a bread wheat, with 2*n* = 42 chromosomes, red hard grains, an alternative growth habit, and good bread quality.

### 2.2 Chemicals

High‐purity analytical standards such as Trolox (6‐hydroxy‐2,5,7,8‐tetramethylchroman‐2‐carboxylic acid), 2‐hydroxycinnamic, 4‐hydroxybenzoic, caffeic, ferulic, gallic, protocatechuic, sinapic, syringic, and vanillic acids were obtained from Sigma‐Aldrich, Steinheim, Germany. Whereas DPPH (2,2‐diphenyl‐1‐picrylhydrazyl) and ρ‐coumaric and chlorogenic acids were purchased from the European Pharmacopia Reference Laboratory Germany, Toronto Research Chemicals Inc. Canada, and HWI Pharma Services, GmbH Rülzheim, Germany, respectively.

HPLC‐grade ethyl acetate, methanol, sodium hydroxide, potassium persulfate, sodium carbonate, Folin–Ciocalteu reagent, and ABTS (2,2′‐azinobis (3‐ethylbenzothiazoline‐ 6‐sulfonic acid)) were purchased from Sigma‐Aldrich, Steinheim, Germany. Analytical grade hydrochloric acid was obtained from Merck, Darmstadt, Germany, whereas ethanol and diethyl ether were supplied by Isolab Laborgeräte, GmbH Wertheim, Germany.

### 2.3 Sampling and Pretreatment

After harvesting, hulled wheat kernels underwent manual dehulling and cleaning in order to remove any foreign objects or fractured seeds. Then cleaned wheat grains were milled to obtain a fine whole grain powder (60 mesh size) using a laboratory‐scale flour mill (Brabender Quadrumat Junior laboratory mill, South Hackensack, NJ, USA), following the AACC No: 26–50 method (AACC 2000). Finally, each whole grain flour sample was vacuum‐packed in moisture‐proof plastic bags and kept at −20°C. In addition, AACC No: 08–01 method (AACC 2000) was employed to calculate the ash contents (%) of whole grain flour in all wheat samples.

### 2.4 Analysis of Phenolic Compounds

#### 2.4.1 Extraction

The extraction of soluble (free and conjugated), insoluble bound phenolic contents, individual phenolic acids, and total flavonoid contents was carried out on whole grain wheat samples according to the method reported by Moore et al. (2005). Briefly, the soluble free and conjugated phenolic compounds were extracted from the whole grain flour samples by using a water/methanol/acetone (6:7:7, v/v/v) solvent mix. Then, the fractions containing soluble conjugated and insoluble bound phenolic compounds were hydrolyzed with 2 N NaOH, and the pH was adjusted to 1.5–2 with the help of 6 N HCl. Each individual sample underwent extraction four times, using a solvent mix containing ethyl acetate and diethyl ether (1:1, v/v). Following centrifugation (6100 × g for 5 min), all supernatants were combined and evaporated at 35°C using pure nitrogen and then redissolved into 1 mL of methanol and Milli‐Q water (3:7, v/v) solution mix. The obtained extracts were passed through Polytetrafluoroethylene (PETE) syringe filter (0.22 μm pore size) and kept at −20°C.

#### 2.4.2 Phenolic Contents

The Folin‐Ciocalteau method has been employed to determine the soluble (free and conjugated) and insoluble bound phenolic contents in all wheat varieties (Serpen et al. 2008). However, total phenolic contents (TPC) were calculated by the addition of soluble and insoluble phenolic contents. The coefficient of determination (*R* ^2^) for gallic acid standard was calculated as 0.999 (Figure S2). Quantitative results were expressed in mg of gallic acid equivalent (GAE) per kilogram of whole grain on dry matter (dm) basis.

#### 2.4.3 Phenolic Acid Composition

A previously reported well‐refined method by Irakli et al. (2012) was adopted for the analysis of phenolic acid composition using HPLC‐DAD. A Nucleosil 100–5 C18ec (25 cm length, 5 μm particle size, to be consistent with 0.56 cm I.D) column from Macherey‐Nagel, Düren, Germany, was utilized for chromatographic isolation of individual phenolic acids. The process was conducted at 30°C with a 20 μL sample injection using an Agilent HPLC 1100 system (Agilent Technologies, Waldbronn, Germany) equipped with a DAD detector. The mobile phase is composed of pure methanol (A) and acetic acid:Milli‐Q water (1:99, v/v) solution (B). The elution was carried out at 1.3 mL/min flow rate with the following gradient: 90% to 80% B (10 min), 80% to 75% B (10 min), 75% to 65% B (10 min), 65% to 35% (10 min), and finally returning to 90% B for 10 min to re‐equilibrate the column.

The chromatograms for 4‐hydroxybenzoic, vanillic, and protocatechuic acids were detected at 254 nm; for 2‐hydroxycinnamic, gallic, and syringic acids at 280 nm; and for caffeic, chlorogenic, ferulic, ρ‐coumaric, and sinapic acids at 320 nm. The coefficient of determination (*R* ^2^) for caffeic, chlorogenic, 4‐hydroxybenzoic, 2‐hydroxycinnamic, protocatechuic, ρ‐coumaric, gallic, ferulic, sinapic, syringic, and vanillic acids was observed as ≥ 0.99 (Table S2). The identification of phenolic acids in wheat samples was achieved by comparing the retention time and absorption spectra of peaks with those of the reference standards. The quantification was carried out by comparing the calibration curves built for each of the phenolic acids, identified in the wheat samples, and the results of the individual phenolic acids were presented in mg/kg dm of whole grain wheat flour.

#### 2.4.4 Flavonoid Contents

The total flavonoid contents (TFC) in the indigenous wheat varieties were measured using a previously reported colorimetric method (Serpen et al. 2008). In brief, an appropriate dilution of the extracts (200 μL) was mixed with 100 μL of 5% NaNO_2_ for 5 min, and a flavonoid‐aluminum complex was formed by adding 1 mL of 10% AlCl_3_. After adding 500 μL of 1 N NaOH to the mixture, centrifugation was achieved at 10,000 × *g* for 5 min at 25°C. After 15 min of incubation period, the absorbance of the supernatant was measured using a UV/Vis spectrophotometer at a wavelength of 510 nm. The coefficient of determination (*R* ^2^) for catechin standard was calculated as 0.99 (Figure S2). The results were reported as mg of catechin equivalent (CE) per kg dm of whole wheat grain.

### 2.5 Total Antioxidant Capacity

A direct QUENCHER‐based approach was used to determine the total antioxidant capacity of the whole wheat grain samples (Serpen, Gökmen, and Fogliano 2012). In particular, ABTS•+ and DPPH• radical‐based assays were employed for the measurement of the total antioxidant capacity. A stock solution of ABTS•+ radical was prepared at a final concentration of 7 mmol/L ABTS•+ radical and 2.45 mmol/L potassium persulfate (K_2_S_2_O_8_). To achieve this, 38.41 mg ABTS•+ in 5 mL Milli‐Q water and 6.615 mg K_2_S_2_O_8_ in 5 mL Milli‐Q water were dissolved separately. Then both solutions were combined to make a stock solution and left in the dark for 15 h at room temperature before being used to prepare a working solution. Similarly, the stock solution of the DPPH• radical was prepared in a final concentration of 0.5 mmol/L. For this, 20 mg of the DPPH• radical was first dissolved in 50 mL of ethanol, and then the solution was further diluted with an additional 50 mL Milli‐Q water. Finally, the inhibition percentage of ABTS•+ and DPPH• radicals was determined and transformed to Trolox equivalent antioxidant capacity (TEAC) with the help of Trolox reference standard. Standard calibration curves were determined by mixing 0.1 mL of a standard solution of each concentration (100–600 ppm) with 10 mL of ABTS•+ and DPPH• radicals working solutions separately, and their absorbances were measured separately at 734 and 520 nm, respectively. As a result, the coefficient of determination (*R* ^2^) for ABTS•+ and DPPH• radicals was calculated as 0.997 and 0.999, respectively (Figure S2). The results were reported as mmol TEAC per kg of whole wheat grain on dm basis.

### 2.6 Statistical Analysis

The statistical analysis was performed using the IBM SPSS Statistics package (version 26.0, IBM Corporation, USA). A one‐way analysis of variance (ANOVA‐I), followed by Duncan's post hoc test, was performed to determine the statistically significant variation (*p* < 0.05) among the results. The relationship between phenolic acids, TFC, and antioxidant capacity has been explained by using Heatmap and PCA biplot analyses after normalizing the data to a range of −2 to 2. Specifically, heatmap was generated by the GraphPad Prism v. 9.0 (GraphPad Software, MA, USA), and PCA was plotted using OriginPro software version 2023b (OriginLab Corporation, MA, USA).

## 3 Results and Discussion

### 3.1 Phenolic Compounds

#### 3.1.1 Phenolic Contents

**FIGURE 1:**
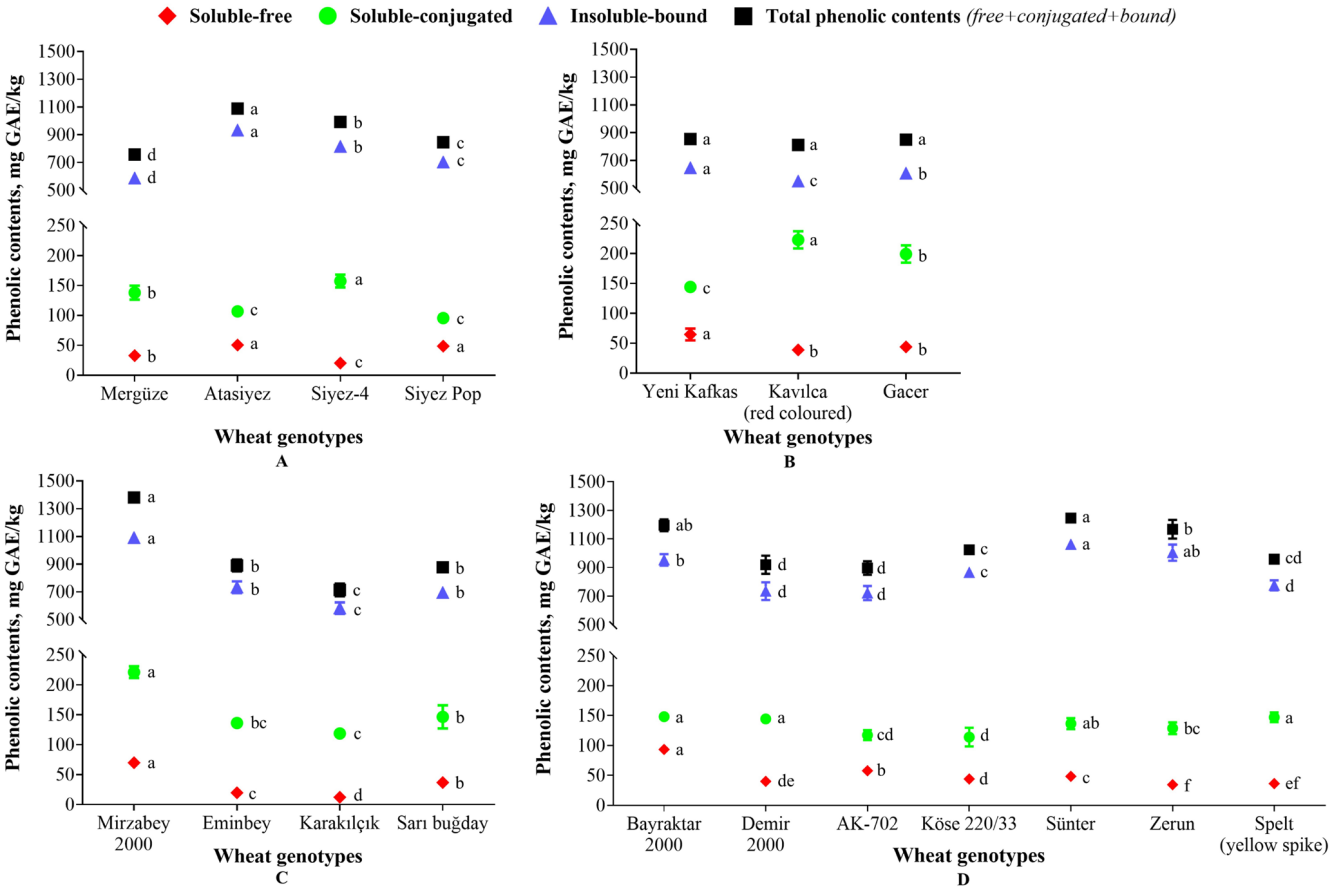
Phenolic contents in (A) monococcum, (B) dicoccum, (C) durum, and (D) aestivum wheat genotypes. Values with different lowercase letters in the same results are statistically different (*p* < 0.05), (Mean ± SD, *n* = 4).

Figure 1 demonstrates the findings regarding the amounts of soluble free, soluble conjugated, insoluble bound, and total phenolic content (TPC) among all wheat genotypes. In monococcum genotype (Figure 1A), the recently commercialized Atasiyez variety contains significant amounts of total, insoluble bound, and soluble free phenolic contents (1089.38, 932.37, and 50.43 mg GAE /kg, respectively), with the exception of soluble conjugate phenolics. However, Siyez‐4 displayed the highest amount of 157.28 mg GAE/kg for soluble conjugated phenolics. In addition, Mergüze wheat exhibited the lowest TPC of 757.49 mg GAE/kg, which mainly consists of soluble conjugated phenolic compounds. The TPC values in all dicoccum varieties were observed to be non‐significantly different (*p* > 0.05), as shown in Figure 1B. Yeni Kafkas wheat, which has recently been commercialized, exhibits elevated levels of insoluble bound and soluble free phenolics, measuring 646.24 and 64.54 mg GAE /kg, respectively. The Kavılca (red colored) wheat has a considerably higher concentration of soluble conjugated phenolics (222.65 mg/kg) within the dicoccum genotype (*p* < 0.05).

The Mirzabey 2000 wheat variety in durum genotype exhibited remarkably high levels of total (1381.38 mg/kg), insoluble bound (1090.39 mg/kg), soluble conjugated (221.25 mg/kg), and soluble free (69.74 mg/kg) phenolic contents compared to other wheat varieties (Figure 1C). In a similar manner, high amounts of soluble free phenolic contents (93.42 mg/kg) have been detected in Bayraktar 2000 wheat of aestivum genotype (Figure 1D). Bayraktar 2000, Demir 2000, Zerun, and Spelt (yellow spike) also showed significantly higher levels of soluble conjugated phenolic compounds compared to Köse 220/33 and AK‐702 wheat varieties. In addition, significant levels of insoluble bound phenolics were found in the Sünter (1061.28 mg/kg) and Zerun (1004.28 mg/kg) varieties. However, the indigenous Sünter variety exhibited the maximum TPC of 1246.30 mg GAE/kg dm when compared with other varieties of aestivum genotype.

Overall, TPC contents were found in the range of 757.49 and 1089.38 mg in monococcum, 811.39 and 854.77 mg in dicoccum, 713.92 and 1381.38 mg in durum, and 897.05 and 1246.30 mg GAE/kg dm in aestivum genotypes. Similar to our findings, comparable results for total phenolic contents were reported in various European wheat landraces (Lachman et al. 2011). In a study, Zhang et al. (2012) reported the average bound phenolic concentration of 661 μg/g of dm in 37 different Chinese winter wheat cultivars, which is also consistent with our results. Additionally, the bound phenolic contents of the durum and aestivum genotypes in our study are consistent with findings published in earlier literature (Adom and Liu 2002; Dinelli et al. 2009; Irakli et al. 2012). In contrast to our findings, Serpen et al. (2008) reported that emmer ( *T. dicoccum* ) exhibited higher TPC values than einkorn ( *T. monococcum* ) wheat, which varied from 5.38 to 8.58 and 2.55 to 4.73 μmol GAE/g of whole wheat flour, respectively. Furthermore, investigations carried out on purple colored wheat and synthetic hexaploid wheat have shown comparatively higher levels of phenolic contents than the wheat varieties examined in our study (Shamanin et al. 2022, 2023). This discrepancy may be the result of possible differences in the selected wheat cultivars.

In addition, the total phenolic contents of wheat varieties can also be influenced by various other climatic factors such as irrigation, soil, and fertilizer. In a study, Ma et al. (2015) studied the effect of different amounts of nitrogen fertilizers on total phenolic content in Chinese wheat varieties at Zhengzhou and Wenxian locations and reported the highest TPC values of 1451.5 and 1397.9 μg/g, respectively, with 300 kg N/ha, compared to no nitrogen application. However, the highest total soluble and insoluble phenolic contents, with low nitrogen concentration (90 kg N/ha) and high phosphate application (209 kg P_2_O_5_/ha), respectively, were reported in pigmented wheat cultivars after studying the combined effects of nitrogen and phosphate‐based fertilizers (Ma et al. 2018).

#### 3.1.2 Phenolic Acid Composition

Seven individual phenolic acids such as 4‐hydroxybenzoic, caffeic, sinapic, syringic, ρ‐coumaric, ferulic, and vanillic acids were identified in all wheat varieties by comparing the peaks of 11 different pure standard phenolic acids using HPLC‐DAD (Agilent 1100) system (Figure S3). The phenolic acid composition within all wheat varieties was found comparable, with the exception of caffeic acid, which was primarily detected in the monococcum group at trace levels (Table 2). The literature indicates that the concentration of caffeic acid in whole wheat grains is quite low and therefore does not have a significant effect on the overall composition of phenolic acids (Hefni, Amann, and Witthöft 2019). Ferulic and sinapic acids were the most prominent phenolic acids, while other phenolic acids, such as 4‐hydroxybenzoic, caffeic, ρ‐coumaric, syringic, and vanillic acids, were present in lower amounts. The mean distribution of phenolic acids in the monococcum, dicoccum, durum, and aestivum genotypes is also represented in a Pie chart (Figure S4). In monococcum genotype, ferulic, sinapic, and ρ‐coumaric acids consisted of 62%, 27%, and 7% of total phenolic acids, respectively. However, compared to monococcum, sinapic acid increased to 29%, and ρ‐coumaric acid decreased to 5%, while ferulic acid remained unchanged (62%) in the dicoccum genotype. On the other hand, ferulic acid decreased from 67% (durum) to 63% (aestivum), while sinapic acid increased from 26% (durum) to 29% (aestivum), and ρ‐coumaric acid (3%) was not changed in both genotypes.

**TABLE 2:** Phenolic acid profile of wheat genotypes (mg/kg dm of whole grain flour).*

Wheat genotypes	4‐hydroxybenzoic acid	Vanillic acid	Caffeic acid	Syringic acid	ρ‐coumaric acid	Ferulic acid	Sinapic acid	Total phenolic acids
*Monococcum*								
Mergüze	4.96 ± 0.19^b^	12.62 ± 0.83^a^	n.d.	6.91 ± 0.58^a^	52.87 ± 3.51^a^	412.22 ± 13.43^b^	182.93 ± 8.13^b^	672.51 ± 26.36^b^
Atasiyez	5.38 ± 0.07^a^	11.62 ± 0.16^b^	15.32 ± 0.43^b^	6.35 ± 0.16^b^	45.00 ± 2.36^b^	440.45 ± 5.70^a^	196.66 ± 5.46^a^	720.77 ± 9.92^a^
Siyez‐4	4.37 ± 0.16^c^	12.25 ± 0.08^ab^	17.56 ± 1.19^a^	7.36 ± 0.16^a^	51.15 ± 2.71^a^	421.92 ± 9.19^b^	173.96 ± 2.35^b^	688.56 ± 10.60^b^
Siyez population	5.10 ± 0.23^b^	11.76 ± 0.32^b^	15.50 ± 0.32^b^	6.39 ± 0.20^b^	40.45 ± 4.26^b^	424.56 ± 10.67^b^	177.99 ± 10.15^b^	681.73 ± 25.92^b^
Average	4.95 ± 0.16	12.06 ± 0.35		6.75 ± 0.28	47.37 ± 3.21	424.79 ± 9.75	182.88 ± 6.52	690.89 ± 25.84
*Dicoccum*								
Yeni Kafkas	5.29 ± 0.18 ^b^	15.03 ± 0.54^b^	14.07 ± 0.15	7.93 ± 0.26^b^	33.60 ± 0.28^a^	421.39 ± 4.16^a^	180.60 ± 3.96^b^	677.92 ± 4.75^a^
Kavılca (red colored)	4.37 ± 0.10^c^	14.15 ± 0.25^c^	n.d.	8.77 ± 0.50^a^	33.07 ± 1.38^a^	409.98 ± 3.99^a^	195.63 ± 2.83^a^	665.98 ± 7.53^a^
Gacer	7.04 ± 0.44^a^	17.06 ± 0.71^a^	n.d.	6.65 ± 0.22^c^	23.85 ± 1.46^b^	351.63 ± 25.86^b^	181.96 ± 11.66^b^	588.18 ± 40.20^b^
Average	5.57 ± 1.18	15.41 ± 1.36		7.79 ± 0.96	30.17 ± 4.80	394.33 ± 34.78	186.06 ± 9.69	644.03 ± 46.79
*Durum*								
Mirzabey 2000	12.44 ± 0.73^a^	16.06 ± 0.83^a^	n.d.	5.81 ± 0.27^d^	28.99 ± 1.48^a^	519.72 ± 25.86^a^	194.79 ± 8.45^c^	777.81 ± 36.36^a^
Eminbey	8.99 ± 0.74^a^	13.03 ± 0.42^b^	n.d.	7.46 ± 0.27^b^	23.28 ± 1.33^a^	553.53 ± 37.63^a^	208.80 ± 7.44^b^	815.08 ± 46.60^a^
Karakılçık	6.26 ± 0.17^c^	15.68 ± 0.37^a^	n.d.	8.40 ± 0.17^a^	23.49 ± 0.18^a^	461.35 ± 7.73^b^	166.65 ± 4.46^c^	681.83 ± 11.73^b^
Sarı bugday	8.13 ± 0.21^b^	15.51 ± 0.33^a^	n.d.	6.66 ± 0.09^c^	22.57 ± 0.79^a^	536.39 ± 14.17^a^	224.93 ± 6.85^a^	814.20 ± 21.56^a^
Average	8.96 ± 2.36	15.07 ± 1.32		7.08 ± 1.01	24.58 ± 2.82	517.75 ± 41.86	198.79 ± 22.97	772.23 ± 62.98
*Aestivum*								
Bayraktar 2000	7.50 ± 0.07^c^	16.59 ± 0.18^cd^	n.d.	10.68 ± 0.25^d^	15.04 ± 0.35^f^	406.66 ± 3.78^d^	167.64 ± 15.53^d^	624.10 ± 19.91^d^
Demir 2000	6.59 ± 0.09^d^	15.53 ± 0.50^d^	14.45 ± 0.37	18.19 ± 0.32^a^	20.59 ± 0.46^de^	510.95 ± 7.75^b^	237.86 ± 1.62^b^	824.17 ± 7.28^b^
AK‐702	7.48 ± 0.28^c^	16.61 ± 0.65^cd^	n.d.	8.64 ± 0.46^e^	23.28 ± 0.52^c^	449.86 ± 14.67^c^	211.46 ± 13.93^c^	717.33 ± 30.31^c^
Köse 220/33	7.81 ± 0.33^b^	18.17 ± 1.49^b^	n.d.	12.56 ± 1.01^bc^	21.63 ± 1.40^d^	514.10 ± 33.68^b^	238.60 ± 22.70^b^	812.88 ± 60.40^b^
Sünter	6.28 ± 0.03^d^	17.13 ± 0.57^bc^	n.d.	12.02 ± 1.22^c^	25.77 ± 0.60^b^	550.65 ± 13.91^a^	267.12 ± 9.16^a^	878.97 ± 24.79^a^
Zerun	8.33 ± 0.17^a^	25.36 ± 0.57^a^	n.d.	13.30 ± 0.20^b^	20.01 ± 1.61^e^	430.69 ± 6.50^c^	237.64 ± 5.67^b^	735.33 ± 1.19^c^
Spelt (yellow spike)	6.51 ± 0.04^d^	13.63 ± 0.59^e^	n.d.	9.96 ± 0.26^d^	27.25 ± 0.24^a^	496.58 ± 9.25^b^	176.70 ± 14.86^d^	730.64 ± 9.95^c^
Average	7.21 ± 0.74	17.57 ± 3.56		12.19 ± 2.97	21.94 ± 3.89	479.93 ± 50.79	219.58 ± 36.22	760.49 ± 83.55

Abbreviation: n.d., not detected.

* Values with different lowercase letters in the same results and individual group are statistically different (*p* < 0.05), mean ± SD, *n* = 4.

The amounts of total phenolic acids in various wheat genotypes varied by the following: 672.51–720.77 mg in monococcum, 588.18–677.92 mg in dicoccum, 681.83–815.08 mg in durum, and 624.10–878.97 mg/kg dm in aestivum genotypes. The Mergüze and Siyez‐4 wheat varieties have shown high levels of ρ‐coumaric and vanillic acids in the monococcum genotype. Furthermore, Mergüze was the only member of the monococcum group that did not show any detectable caffeic acid. The Atasiyez wheat exhibited the highest concentrations of ferulic, sinapic, and 4‐hydroxybenzoic acids, followed by Siyez Population variety in monococcum group. The Kavılca (red colored) and Yeni Kafkas in dicoccum genotype display notable amounts of ferulic, syringic, and ρ‐coumaric acids. Additionally, sinapic acid was found abundant in Kavılca (red colored) variety. Furthermore, Gacer wheat has been discovered to possess significant quantities of vanillic and 4‐hydroxybenzoic acids.

The highest amount of ferulic acid was discovered in the Mirzabey 2000 and Eminbey wheat varieties of durum genotype. In contrast, Karakılçık and Sarı bugday varieties have considerable amounts of syringic and sinapic acids, respectively. In aestivum genotype, Sünter wheat has been found to contain significant quantities of ferulic acid and sinapic acid, whereas caffeic acid was only observed in Demir 2000 variety. Similarly, 4‐hydroxybenzoic and vanillic acids were abundant in Zerun wheat whereas Spelt (yellow spike) variety was found rich in ρ‐coumaric acid.

The composition and quantities of phenolic acids in Turkish wheat varieties were determined to be comparatively similar to those documented in the literature for aestivum and durum genotypes (Irakli et al. 2012). In another study, Shamloo et al. (2017) reported that the phenolic acid contents in various Canadian and Australian wheat varieties ranged from 389.54 ± 32.36 mg/kg dm at 20°C to 1007.61 ± 87.32 mg/kg dm at 30°C, which were comparable to the levels found in Turkish wheat varieties of this study. Similarly, the impact of abiotic stresses such as draught and heat on the phenolic composition of six different Mexican durum wheat cultivars was investigated by Laddomada et al. (2021), and ferulic acid was found as prominent phenolic acid (390.1 to 785.6 μg/g dm), followed by sinapic (29.4–92.3 μg/g dm) and ρ‐coumaric (7.4–38.3 μg/g dm) acids, which is again consistent to the results of our study. The accumulation of ferulic and total phenolic acids was improved under severe drought whereas ρ‐coumaric and syringic acids was enhanced during heat stress. In another study, Kowalska et al. (2022) investigated the effects of various factors such as genotype, climate temperatures, soil types, and fertilizer applications on 12 different winter wheat varieties from eight European locations. The highest average total phenolic acid contents (1016.19 μg/g of grain dry matter) observed in the Spanish wheat variety may be attributed to the hot climate of Spain. In addition, the effects of varying nitrogen application rates on the phenolic acid composition of Chinese elite bread wheat varieties at four different locations were also studied for two years, and it was revealed that ρ‐coumaric, sinapic, and cis‐ferulic acid concentrations were significantly affected by nitrogen levels, whereas trans‐ferulic acid concentration remained unchanged (Tian et al. 2022).

#### 3.1.3 Total Flavonoid Contents

**FIGURE 2:**
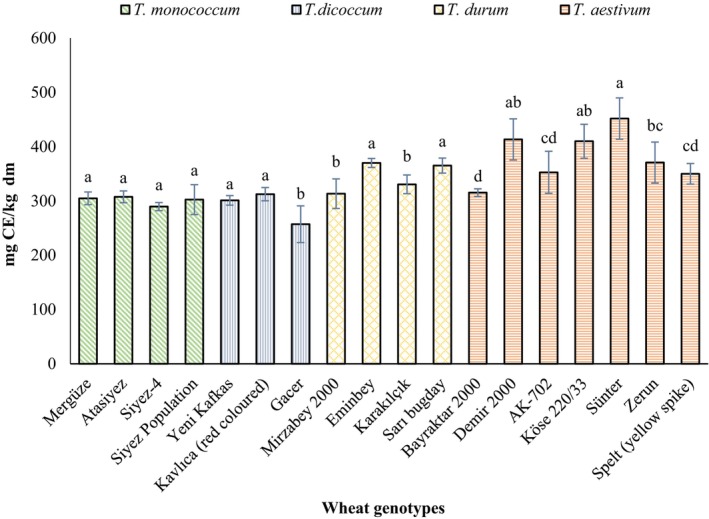
Total flavonoid contents of wheat genotypes. Values with different lowercase letters in the same results and individual group are statistically different (*p* < 0.05), (Mean ± SD, *n* = 4).

The results for total flavonoid contents (TFC) in all wheat genotypes are presented in Figure 2. The distribution of TFC contents was found between 289.54 and 307.52 mg in monococcum, 257.02 and 312.38 mg in dicoccum, 313.25 and 369.86 mg in durum, and 315.27 and 451.63 mg CE/kg dm in aestivum genotypes. The TFC contents reduced from aestivum genotype having the highest mean value of 380.43 mg, followed by durum with a mean value of 344.64 mg to the monococcum genotype with mean value of 301.03 mg, and the dicoccum genotype with the lowest mean value of 290.10 mg CE/kg dm.

The total flavonoid content (TFC) in the monococcum genotype did not show a significant increase (*p* > 0.05). In contrast, there were significant differences in flavonoid levels among the dicoccum, durum, and aestivum genotypes (*p* < 0.05). Eminbey and Kavılca (red colored) wheat varieties showed the highest TFC contents of 369.86 mg and 312.38 mg CE/kg dm in the durum and dicoccum genotypes, respectively. Sünter variety in aestivum genotype showed the higher total flavonoid contents of 451.63 mg compared to 413.26 mg CE/kg dm in commercial Demir 2000 variety. Similar to the results of our study, Shamloo et al. (2017) reported the flavonoid contents in various Canadian and Australian wheat varieties varying from 170.05 ± 2.24 at 20°C to 343.23 ± 3.03 μg rutin equivalent/g dry matter at 30°C. In another study, Sharma et al. (2023) reported 3.36 to 13.44 mg quercetin equivalent/100 g of flavonoid contents in blue, purple, and black colored wheat varieties, which are comparatively lower than the findings of our study. Additionally, Ma et al. (2015) reported the highest TFC contents of 0.66 and 0.53 mg/g in Chinese wheat at the Wenxian and Zhengzhou locations, respectively, following the application of 300 kg N/ha of nitrogen fertilizer. However, our findings differ from those of Dinelli et al. (2009), who reported lower levels of flavonoids in both ancient and modern durum wheat varieties. These variations could be attributed to differences in used wheat varieties, climatic temperature values, and fertilizer applications.

### 3.2 Ash Contents and Total Antioxidant Capacity

The ash content provides a precise measure of the overall mineral composition in food materials. Sezer et al. (2017) demonstrated that ash content has a direct impact on the mineral content, extraction yield, technological properties, and nutritional labeling of wheat flour, serving as a significant indicator of chemical quality. As presented in Table 3, the ash contents of all wheat varieties were observed in the range of 1.19% to 1.80%. In addition, the average ash contents of all wheat genotypes decreased in the following sequence: durum ≥ monococcum > dicoccum > aestivum. Mirzabey 2000, Bayraktar 2000, Atasiyez, and Gacer wheat, which belong to the durum, aestivum, monococcum, and dicoccum genotypes, respectively, were observed to have significantly high ash contents in their respective groups. Similar to our results, the ash contents have been reported in between 1.18% and 1.75% for five different Canadian wheat varieties (Hatcher and Kruger 1997). However, considerably high ash contents, ranging from 1.76% to 1.86%, were observed in different European wheat varieties cultivated between 1992 and 1994 (Bonafaccia et al. 2000).

**TABLE 3:** Ash contents (%) and total antioxidant capacity (mmol TEAC/kg dm) of wheat genotypes.*

Wheat genotypes	Ash contents	ABTS	DPPH
*Monococcum*			
Mergüze	1.41 ± 0.00^c^	20.40 ± 0.35^a^	4.34 ± 0.07^a^
Atasiyez	1.50 ± 0.01^a^	20.73 ± 1.43^a^	4.64 ± 0.18^a^
Siyez‐4	1.45 ± 0.00^b^	21.01 ± 1.01^a^	4.76 ± 0.37^a^
Siyez Population	1.37 ± 0.01^d^	20.24 ± 1.54^a^	4.43 ± 0.23^a^
Average	1.43 ± 0.05	20.60 ± 1.05	4.54 ± 0.27
*Dicoccum*			
Yeni Kafkas	1.25 ± 0.01^c^	20.37 ± 0.50^a^	5.02 ± 0.36^b^
Kavılca (red colored)	1.33 ± 0.01^b^	20.50 ± 0.63^a^	5.22 ± 0.27^b^
Gacer	1.62 ± 0.03^a^	20.61 ± 0.40^a^	5.81 ± 0.19^a^
Average	1.40 ± 0.18	20.49 ± 0.46	5.35 ± 0.43
*Durum*			
Mirzabey 2000	1.80 ± 0.01^a^	30.11 ± 044^a^	4.44 ± 0.26^a^
Eminbey	1.47 ± 0.01^b^	17.15 ± 0.61^a^	4.12 ± 0.32^a^
Karakılçık	1.35 ± 0.01^c^	19.17 ± 0.16^a^	4.11 ± 0.40^a^
Sarı bugday	1.19 ± 0.01^d^	17.10 ± 0.53^b^	4.26 ± 0.20^a^
Average	1.45 ± 0.24	20.95 ± 5.60	4.16 ± 0.33
*Aestivum*			
Bayraktar 2000	1.80 ± 0.02^a^	22.00 ± 0.22^a^	3.74 ± 0.06^bc^
Demir 2000	1.34 ± 0.03^cd^	18.46 ± 0.16^cd^	4.24 ± 0.22^a^
AK‐702	1.46 ± 0.04^b^	19.12 ± 0.64^bcd^	4.20 ± 0.23^a^
Köse 220/33	1.35 ± 0.02^c^	19.33 ± 0.52^bc^	3.37 ± 0.37^c^
Sünter	1.25 ± 0.01^de^	19.48 ± 0.78^b^	4.20 ± 0.29^a^
Zerun	1.22 ± 0.01^e^	18.23 ± 0.28^d^	3.66 ± 0.29^bc^
Spelt (yellow spike)	1.30 ± 0.08^cde^	20.37 ± 0.28^a^	4.14 ± 0.23^ab^
Average	1.39 ± 0.19	19.57 ± 1.27	3.94 ± 0.39

* Values with different lowercase letters in the same results and individual group are statistically different (*p* < 0.05), mean ± SD, *n* = 3.

The results of total antioxidant capacities determined by ABTS and DPPH assays are reported in Table 3. The ABTS results indicated that the Trolox equivalent antioxidant capacities ranged from 20.24 to 21.01 mmol in monococcum, 20.37 to 20.61 mmol in dicoccum, 17.10 to 30.11 mmol in durum, and 18.23 to 22.00 mmol TEAC/kg dm in aestivum wheat genotypes (Table 3). However, the DPPH‐based TEAC values varied from 4.34 to 4.76 mmol in monococcum, 5.02 to 5.81 mmol in dicoccum, 4.11 to 4.44 mmol in durum, and 3.37 to 4.24 mmol TEAC/kg dm in aestivum genotypes (Table 3).

The variations in total antioxidant capacity among different varieties of wheat exhibited a similar pattern when comparing the two different assays (ABTS and DPPH). However, the high TEAC values obtained from the ABTS assay than from the DPPH assay can be attributable to the unique scavenging abilities of respective radicals against different antioxidant groups. The ABTS radical is hydrophilic and useful in evaluating the electron donating potential of a broader range of molecules. However, DPPH radical measures the hydrogen transfer capacity of antioxidants but exhibits less preference towards polar metabolites due to its hydrophobic nature. ABTS and DPPH assays use different scavenging radicals and, therefore, their results cannot be directly compared with each other (Serpen et al. 2008).

In our study, we observed that the total antioxidant capacity values were not significantly (*p* > 0.05) different among monococcum genotypes in each antioxidant assay. Among all wheat varieties, Mirzabey 2000 and Gacer displayed the highest antioxidant capacity in durum and dicoccum genotypes, for ABTS•+ and DPPH• radicals, respectively. The observed differences in antioxidant values between the ABTS and DPPH assays can be attributed to the interaction of the radicals with both soluble and bound phenolics. The ABTS assay predominantly measures the interaction of radicals with bound phenolics, which explains why Mirzabey 2000 having high levels of bound phenolics, showed high TEAC values. On the other hand, DPPH assay primarily interacts with soluble phenolics, therefore Gacer being rich in soluble phenolics, exhibited high antioxidant values. Similarly, Bayraktar 2000 and Demir 2000 varieties in aestivum genotype have been observed with the highest total antioxidant capacity in ABTS and DPPH assays, respectively.

The DPPH‐based antioxidant capacity values of Turkish bread and durum wheat varieties were reported to vary from 3.59 to 4.64 μmol Trolox Equivalent/g flour (Menteş Yılmaz, Bakkalbaşı, and Ercan 2018), which aligns with the DPPH results for the wheat varieties investigated in our study. In the same study, ABTS‐based results were reported between 10.53 and 12.23 μmol TE/g flour, which are lower than our findings. Yiğit and Erekul (2023) also reported slightly higher antioxidant values (varying from 11.89% to 26.33% of DPPH inhibition) for various Turkish bread wheat varieties. Similar to our findings, Serpen et al. (2008) have reported the ABTS‐based total antioxidant capacity ranging from 16.92 to 20.64 mmol and 19.00 to 23.84 mmol TEAC/kg for Turkish monococcum and dicoccum genotypes, respectively. DPPH (% inhibition) values of Indian bread wheats from different agro‐climatic zones were determined between 6.0% and 25.0%. However, the ABTS assay results, which ranged from 2.0 to 10.0 μmol TE/g, were much lower than our findings (Narwal et al. 2014). On the other hand, several studies have reported that pigmented wheat varieties have significantly higher antioxidant activities compared to conventional ones (Sharma et al. 2023; Tian and Li 2018).

Furthermore, ABTS and DPPH assays have been considered to measure the antioxidant capacities in different wheat‐based products. The effects of water‐soluble polysaccharide of cress seeds were investigated on the antioxidant activities of cake formulations by using ABTS and DPPH assays (Ben Slima et al. 2022). In another study, Giuffrè et al. (2023) utilized ABTS•+ and DPPH• radicals to determine the antioxidant capacity of breadsticks over a 12‐month shelf life. Similarly, the antioxidant capacity of gluten‐free muffins prepared with persimmon flour was measured using the DPPH assay during *in vitro* gastrointestinal digestion (Hosseininejad et al. 2022).

### 3.3 Integrated Data Analyses by Heatmap and PCA

**FIGURE 3:**
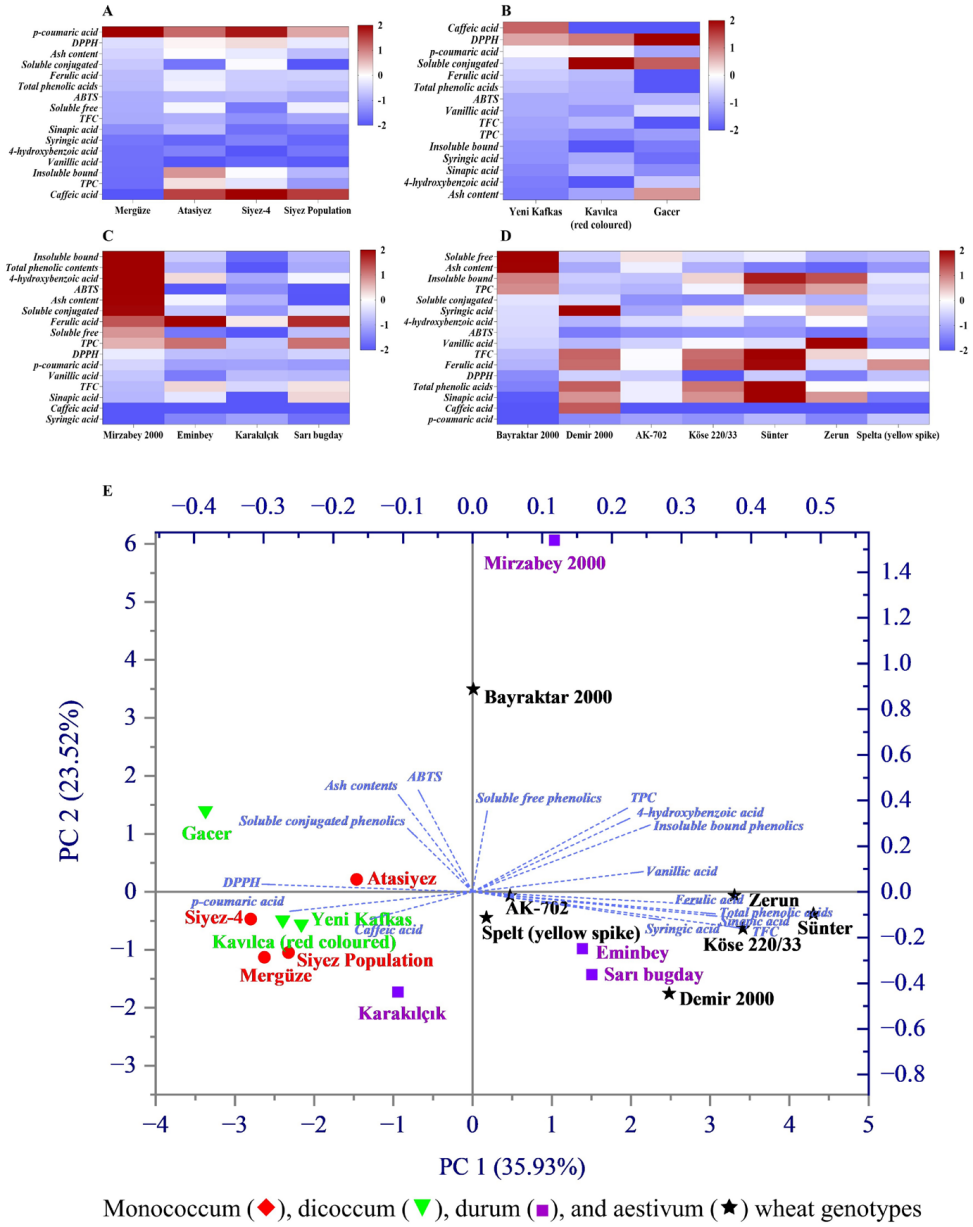
Heatmap and PCA analysis of wheat genotypes.

For better demonstration and understanding of the correlation, a normalized statistical data is presented in the form of Heatmap and PCA biplot. These graphical plots presented a comprehensive insight into the comparison of inter‐ and intra‐wheat genotypes, as presented in Figure 3.

#### 3.3.1 Heatmap Analysis

Heatmaps were generated using normalized data for each individual result, including the ash contents, ABTS, DPPH, TFC, and TPC with their individual phenolic acid composition. As indicated by Figure 3A, Siyez‐4, and Mergüze varieties exhibited higher levels of ρ‐coumaric acid compared to other wheat varieties in monococcum group. On the other hand, Atasiyez wheat presented substantial amounts of total, insoluble bound, and soluble free phenolic contents. In dicoccum genotype (Figure 3B), the Yeni Kafkas variety displayed elevated levels of caffeic acid and soluble free phenolic compounds. In contrast, the Gacer and Kavılca (red colored) varieties showed increased levels of soluble conjugated phenolic contents. Additionally, the Gacer wheat demonstrated a superior DPPH‐based antioxidant capacity than other cultivars of the dicoccum genotype.

The Sarı bugday variety in durum genotype was observed with a high level of ferulic acid, which is comparable to that found in the commercial Eminbey wheat (Figure 3C). In addition, both wheat varieties (Sarı bugday and Eminbey) also demonstrated elevated levels of ferulic acid and TFC in comparison to Karakılçık and Mirzabey 2000 varieties. However, the Sünter wheat of the aestivum genotype showed higher levels of sinapic acid, ferulic acid, insoluble bound phenolics, total phenolic acids, and total flavonoid compounds compared to all other wheat varieties, as shown in Figure 3D. Zerun variety also displayed a comparable profile to Sünter wheat in terms of insoluble phenolic contents, although it exhibited greater amounts of 4‐hydroxybenzoic and vanillic acids. In commercial wheat varieties, Demir 2000 was observed to be rich in caffeic and syringic acids, whereas Bayraktar 2000 showed high values of stearic acid, soluble phenolic, and bound phenolic contents.

#### 3.3.2 PCA Analysis

A PCA biplot analysis was also conducted to identify similarities among various wheat varieties. PC1 and PC2 accounted for 35.93% and 23.52% of the variance, respectively, as illustrated in Figure 3E. The separation of the wheat varieties in the PCA graph is based on the differences in the results of the total antioxidant capacity, ash contents, and phenolic compounds along with individual phenolic acids. Wheat varieties on the positive side of the PC1 in the score plot belong to the durum (except Karakılçık) and aestivum (except Bayraktar 2000) genotypes, while the negative side of PC1 represents the monococcum and dicoccum genotypes. Similarly, both positive and negative sides of PC2 show the distribution of wheat varieties belonging to the monococcum, dicoccum, durum, and aestivum genotypes, as was expected due to their different genetic properties.

The overall PCA results showed the highest values for both Mirzabey 2000 and Bayraktar 2000, which belong to the commercial wheat varieties in durum and aestivum genotypes, respectively. However, indigenous wheat varieties belonging to aestivum genotype exhibited rich phenolic contents. A good correlation between the ash contents, soluble phenolics, and antioxidant capacity was observed for the monococcum and dicoccum genotypes. Monococcum and dicoccum genotypes displayed higher antioxidant capacities than indigenous durum and aestivum genotype. Whereas the durum and aestivum wheat varieties exhibited higher TFC and TPC, which could be related to increased proportion of individual phenolic acids. The interpretations of the results obtained from the principal component analysis aligned with the conclusions drawn from the Heatmap analysis, indicating a correlation between antioxidant capacity and all phenolic compounds.

## 4 Conclusion

The antioxidant capacity, phenolic acid, and flavonoid contents of Turkish indigenous wheat varieties involved in on‐form conservation program were found to be comparable to those of commercial wheat varieties (Mergüze, Yeni Kafkas, Eminbey, and Demir 2000). The high antioxidant values of the indigenous varieties are also correlated with their abundant soluble phenolics and ash contents in respective genotypes. The findings suggested that the indigenous wheat varieties, that is, Siyez‐4, Karakılçık, AK‐702, Köse 220/33, Sünter, and Spelt (yellow spike) have great potential for being registered as commercial varieties and can be incorporated into future breeding initiatives. Furthermore, these results will be used for educational purposes and will encourage local farmers to promote the ongoing conservation of indigenous wheat varieties. Similarly, their existence plays a vital role in generating new prospects for commercial cultivation of indigenous wheat varieties in Turkey and their application in functional food products. It can be concluded that the successful results of this study have the potential to increase the importance of these indigenous wheat varieties in terms of their bioactive characteristics. Moreover, the methodology utilized in this study provides a pragmatic research approach that can be expanded to investigate other indigenous and global varieties.

## Author Contributions

**Muhammad Usman Akram:** data curation (lead), formal analysis (lead), investigation (lead), methodology (lead), software (lead), validation (lead), visualization (lead), writing – original draft (lead), writing – review and editing (lead). **Ayşegül Bilge Uğuz:** formal analysis (supporting), methodology (supporting). **Umran Uygun:** conceptualization (equal), data curation (equal), funding acquisition (equal), project administration (equal), resources (equal), supervision (equal), validation (equal), writing – original draft (equal), writing – review and editing (equal). **Ayten Salantur:** conceptualization (equal), project administration (equal), resources (equal). **Remziye Yilmaz:** conceptualization (equal), data curation (equal), funding acquisition (equal), project administration (equal), resources (equal), supervision (equal), writing – review and editing (equal).

## Conflicts of Interest

The authors declare no conflicts of interest.

## Supporting information


**Table S1.** Monthly average temperature (°C) and precipitation (mm) values of the İkizce location (2019–2020).
**Table S2.** Retention time (Rt), wavelength (nm), regression values (*R* ^2^), LOD and LOQ values for phenolic acids standards mix (1–60 μg/mL) by HPLC‐DAD.
**Figure S1.** Images of Turkish indigenous wheat varieties (both with and without hulled).
**Figure S2.** Spectrophotometric calibration for standards of gallic acid (TPC), catechin (TFC) and Trolox (ABTS•+ and DPPH• scavenging radicals).
**Figure S3.** HPLC chromatograms for phenolic acids in (a) analytical standard mixture and (b) Demir 2000 wheat.
**Figure S4.** Mean distribution of phenolic acids in wheat genotypes.

## Data Availability

The data used in this study will be available from the corresponding authors upon reasonable request.
