# Supplementary material for: Investigation of Phenolic Composition and Antioxidant Capacities in Selected Turkish Indigenous Wheat Varieties
Source: Food Sci Nutr. 2025 Jan 31;13(2):e4614. doi: 10.1002/fsn3.4614 (PMC11782914; doi:10.1002/fsn3.4614)
Supplement: Supplementary file 1 — Table S1. Monthly average temperature (°C) and precipitation (mm) values of the İkizce location (2019–2020). Table S2. Retention time (Rt), wavelength (nm), regression values (R 2), LOD and LOQ values for phenolic acids standards mix (1–60 μg/mL) by HPLC‐DAD. Figure S1. Images of Turkish indigenous wheat varieties (both with and without hulled). Figure S2. Spectrophotometric calibration for standards of gallic acid (TPC), catechin (TFC) and Trolox (ABTS•+ and DPPH• scavenging radicals). Figure S3. HPLC chromatograms for phenolic acids in (a) analytical standard mixture and (b) Demir 2000 wheat. Figure S4. Mean distribution of phenolic acids in wheat genotypes. [file FSN3-13-e4614-s001.docx]

**Investigation of phenolic composition and antioxidant capacities in selected Turkish indigenous wheat varieties**

Muhammad Usman AKRAM^1^, Ayşegül Bilge UĞUZ^1^, Umran UYGUN^1^, Ayten SALANTUR^2^, Remziye YILMAZ^1^*

*^1^Faculty of Engineering, Department of Food Engineering, Hacettepe University, Ankara, Turkey*

*^2^Ministry of Agriculture and Forestry, Field Crops Central Research Institute, Ankara, Turkey*

*Corresponding author: [remziye@hacettepe.edu.tr](mailto:remziye@hacettepe.edu.tr)

# SUPPLEMENTARY MATERIAL

**Standard Agronomic Climatic Conditions**

**Supplementary Table S1.** Monthly average temperature (°C) and precipitation (mm) values of the İkizce location (2019-2020).

| **Months** | **2019-2020**  Temperature (°C) | **Long-term average (min. 30 years)**  Temperature (°C) | **2019-2020** Precipitation (mm) | **Long-term average (min. 30 years)**  Precipitation (mm) |
| --- | --- | --- | --- | --- |
| October | 13.39 | 11.5 | 23.4 | 22.7 |
| November | 6.96 | 5.7 | 31.8 | 29.1 |
| December | 1.49 | 0.9 | 50.8 | 37.7 |
| January | -1.5 | -0.9 | 28.6 | 36.3 |
| February | 3.42 | 1 | 38.7 | 34 |
| March | 6.25 | 5.1 | 13.8 | 35.7 |
| April | 8.77 | 9.7 | 28.6 | 40.2 |
| May | 13.74 | 14.4 | 47.8 | 46.9 |
| June | 17.9 | 18.1 | 27.0 | 35.7 |
| **Total** |  |  | **290.5** | **318.3** |

**Phenolic Acid Composition by HPLC-DAD**

Supplementary Table S2. Retention time (R_t_), wavelength (nm), regression values (R^2^), LOD and LOQ values for phenolic acids standards mix (1 - 60 µg/ml) by HPLC-DAD.

| **Phenolic acids** | **Retention time (R_t_)** | **Wavelength (nm)** | **Calibration curves^a^** | **R^2^** | **LOD^b^ (µg/mL)** | **LOQ^c^ (µg/mL)** |
| --- | --- | --- | --- | --- | --- | --- |
| **Gallic acid** | 5.397 | 280 | y = 38.565x - 42.452 | 0.9994 | 1.952 | 5.914 |
| **Protocatechuic acid** | 9.698 | 254 | y = 45.012x – 9.9821 | 0.9999 | 0.876 | 2.654 |
| **4-hydroxybenzoic acid** | 15.407 | 254 | y = 85.434x – 1.9622 | 0.9999 | 0.705 | 2.137 |
| **Chlorogenic acid** | 18.257 | 320 | y = 19.711x – 19.912 | 0.9992 | 2.148 | 6.510 |
| **Vanillic acid** | 19.526 | 254 | y = 43.100x – 3.8192 | 0.9999 | 0.665 | 2.014 |
| **Caffeic acid** | 20.483 | 320 | y = 41.384x – 22.642 | 0.9997 | 1.265 | 3.833 |
| **Syringic acid** | 22.688 | 280 | y = 46.564x – 3.0676 | 0.9999 | 0.582 | 1.763 |
| **ρ-coumaric acid** | 29.766 | 320 | y = 72.829x – 4.0491 | 0.9999 | 0.678 | 2.053 |
| **Ferulic acid** | 32.868 | 320 | y = 77.600x – 2.7454 | 0.9999 | 0.659 | 1.997 |
| **Sinapic acid** | 33.650 | 320 | y = 34.755x – 21.806 | 0.9986 | 2.874 | 8.710 |
| **2-hydroxycinnamic acid** | 37.503 | 280 | y = 79.895x – 13.245 | 0.9999 | 0.881 | 2.670 |

**^a^**y = response area & x = standard concentration

**^b^**LOD = Limit of Detection,

**^c^**LOQ = Limit of Quantification.

**Images of Wheat Genotypes**


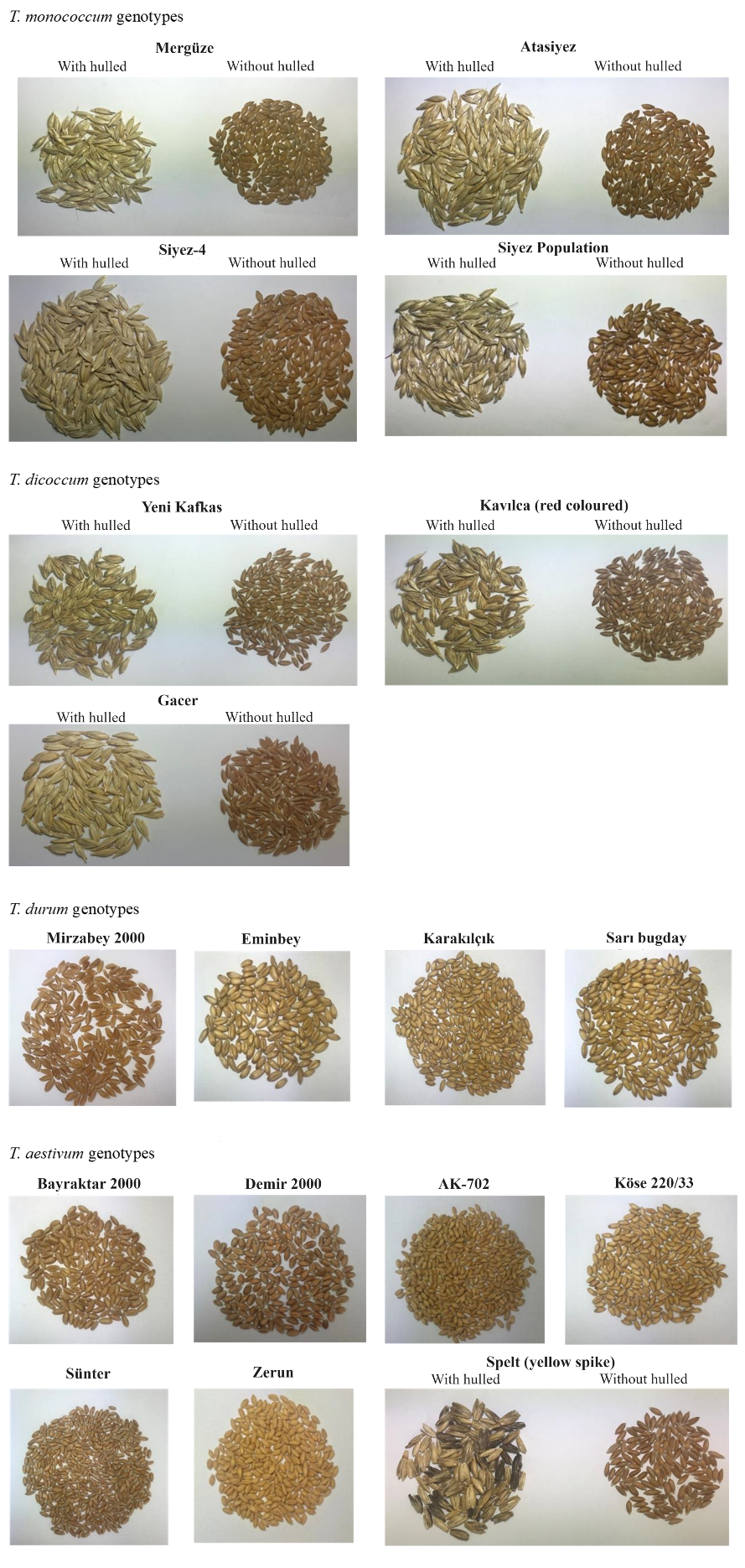


**Supplementary Fig. S1.** Images of Turkish indigenous wheat varieties (with and without hulled).

**Calibration For Phenolic Acids, Flavonoids, and Antioxidant Radicals (ABTS**•+ **and DPPH**•**)**

**
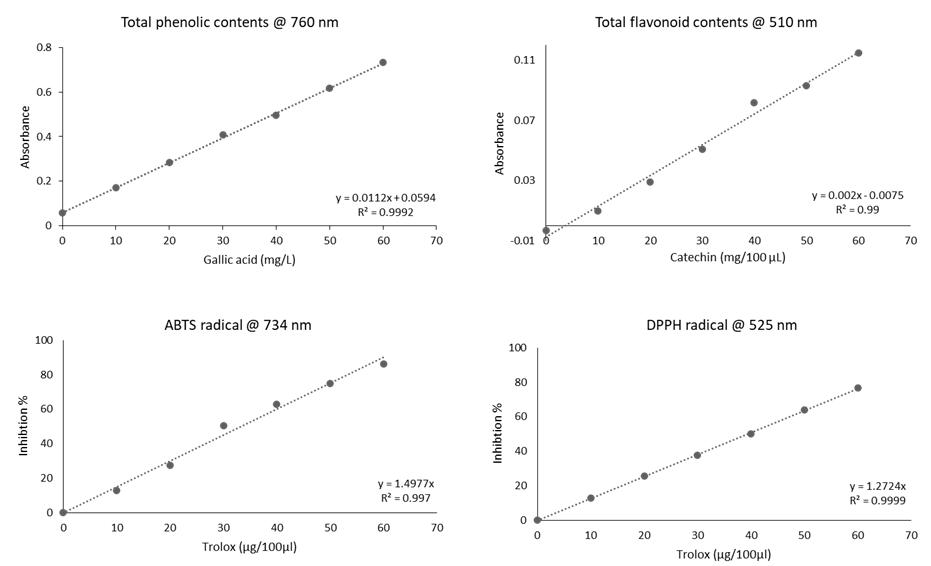
**

Supplementary Fig. S2. Spectrophotometric calibration for standards of gallic acid (TPC), catechin (TFC) and Trolox (ABTS•+ and DPPH• scavenging radicals).

**
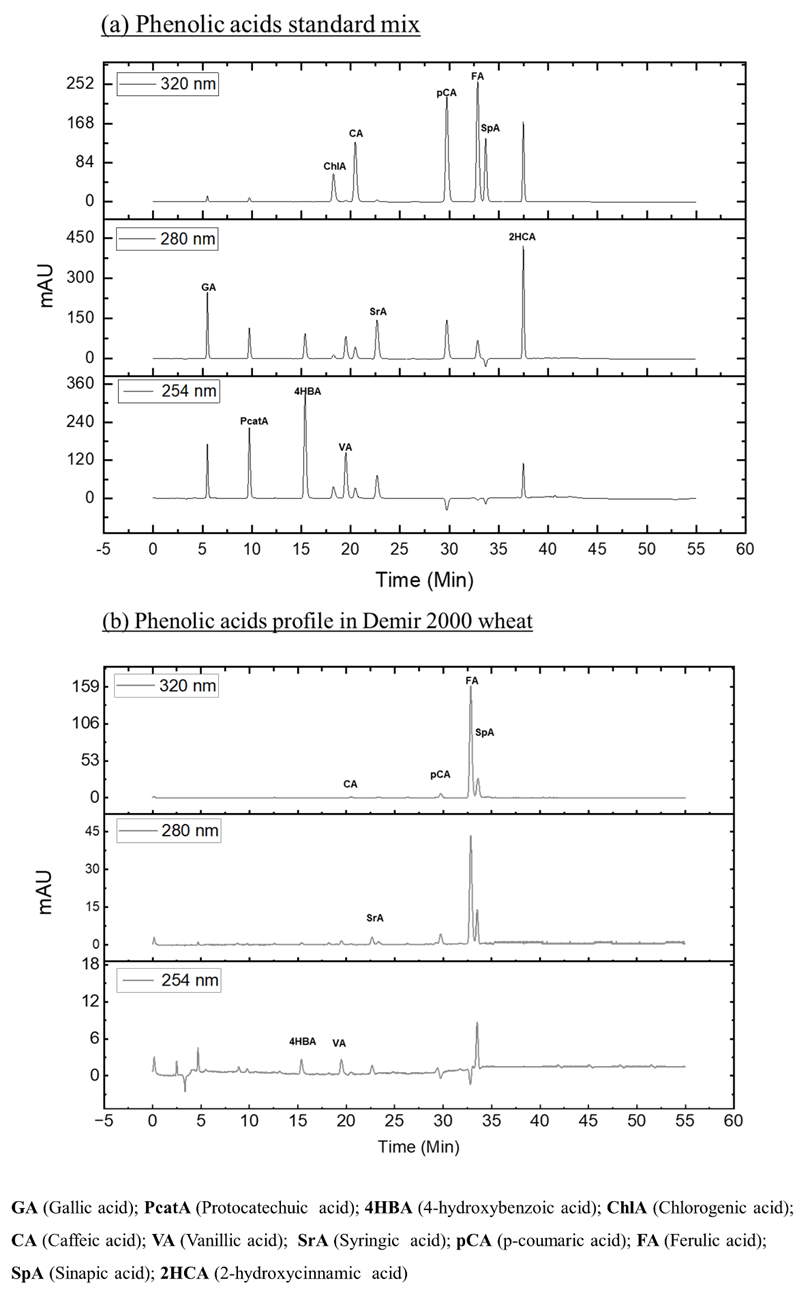
Supplementary Fig. S3.** HPLC chromatograms for phenolic acids in (a) analytical standard mixture and (b) Demir 2000 wheat.


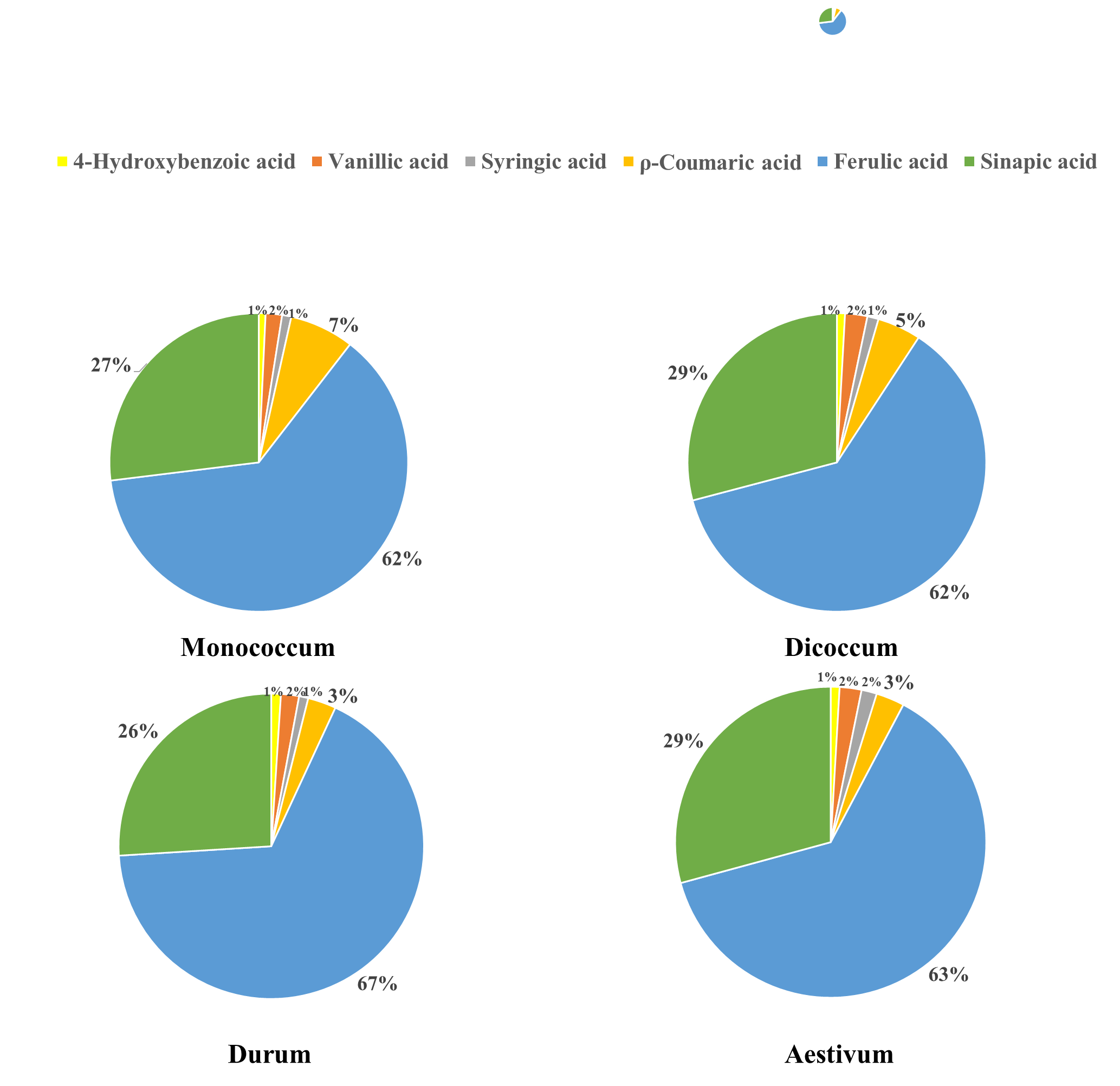


**Supplementary Fig. S4.** Mean distribution of phenolic acids in wheat genotypes.
